# Whole Genome Sequencing of Brucella melitensis Isolated from Patients in Hohhot, China

**DOI:** 10.1128/mra.00032-23

**Published:** 2023-03-27

**Authors:** Shana Chen, Quan Fu, Mandlaa Mandlaa, Qingchun Wang, De Sheng, Saijilahu Saijilahu, Tana Tana, Dezhi Yang

**Affiliations:** a Pharmaceutical Laboratory, International Mongolian Hospital of Inner Mongolia, Hohhot, China; b Department of Clinical Laboratory, Affiliated Hospital of Inner Mongolia Medical University, Hohhot, China; c College of Food Science and Engineering, Inner Mongolia Agricultural University, Hohhot, China; d The Inner Mongolia Autonomous Region Comprehensive Center of Disease Control and Prevention, Hohhot, China; University of Maryland School of Medicine

## Abstract

Brucellosis is a class B infectious disease that is spreading rapidly in Inner Mongolia, China. Investigating the genetics of this disease might provide insights into the mechanism by which the bacteria adapt to their hosts. Here, we report the genome sequence of Brucella melitensis strain BM6144, which was isolated from a human patient.

## ANNOUNCEMENT

A 1-mL sample of wound secretions was inoculated into BacT/ALERT FA Plus medium and placed in a BacT/Alert 3D system at 35°C for 48 to 72 h. For positive detection, 0.2 mL of the culture was spread on a Columbia blood agar plate for isolation. A single colony was selected, and matrix-assisted laser desorption ionization–time of flight mass spectrometry (MALDI-TOF MS) identified a Brucella strain (1). For DNA isolation, strain BM6144 was cultured on Columbia blood agar at 37°C for 48 h, harvested from agar plates with phosphate-buffered saline (PBS), and inactivated at 80°C for 15 min. SDS was used to extract the genomic DNA of the Brucella samples, and a Qubit fluorometer was used to quantify the DNA. Libraries for Oxford Nanopore Technologies (ONT) sequencing were constructed with an insert size of 10 kb. DNA was recovered by using the Blue Pippin system and then repaired. A barcode was added with the PCR-free method for the EXP-NBD104 kit (ONT). The size of the fragments was determined by using an automatic capillary electrophoresis instrument (Advanced Analytical Technologies Inc.). The SQK-LSK109 connection kit was used to link the adapters; subsequently, a 10-kb read library was constructed. Sequencing libraries were generated using the NEBNext Ultra DNA library preparation kit for Illumina (New England Biolabs, USA). The whole genome of BM6144 was sequenced using the Illumina NovaSeq (paired-end 150-bp reads) and ONT PromethION platforms.

The Illumina short reads (*n* = 3,290,108 reads [depth of coverage, 412×]) were used to correct the genome assembly with Pilon v1.22 (2). The raw data were filtered into clean reads using SOAPnuke v2.1.5 (3) to discard reads with proportions of N bases of >10% or proportions of low-quality bases (mass values of ≤20) of >40%. The resulting long-read sequences (*n* = 57,628 reads [*N*_50_, 6,008 bp; depth of coverage, 67×]) were assembled, with a mean read quality score of 11.4. Clean data were assembled using SPAdes v3.10.0 (4) and integrated using CISA v1.3 (5); GapCloser v1.12 (6) was used to optimize the preliminary assembly results. Base calling of the ONT raw data was accomplished using Guppy v6.0.6 (7), and sequences with Q scores of <7 were discarded. Default parameters were used for all software unless otherwise specified.

Hybrid assemblies of Illumina and ONT reads were completed using the Unicycler v0.4.7 pipeline (SPAdes v3.10.0, Racon v1.4.13, and Pilon v1.22) with default parameters (2, 4, 8, 9). The assembly of all of the sequences generated complete circular chromosomes, as well as plasmid contigs. Genomes were rotated to the starting base of the *dnaA* gene. The coverage of core genes was 100% for all genomes. Genome annotation was performed with the NCBI Prokaryotic Genome Annotation Pipeline (PGAP) v6.1 (10). The assembled genome sequence of Brucella melitensis BM6144 was 3,312,177 bp in length. The genome consists of two circular chromosomes, with 1,185,600 bp in chromosome I (G+C content, 57.34%) and 2,126,577 bp in chromosome II (G+C content, 57.15%). These chromosomes possess 3,317 coding gene sequences, 9 rRNA genes, and 55 tRNA genes.

Ethical approval for the collection of isolates was obtained from the Ethics Committee of International Mongolian Hospital of Inner Mongolia (approval number 2021-092).

### Data availability.

This whole-genome sequencing project was deposited in GenBank under accession numbers CP098766.1 and CP098767.1. The raw reads were deposited in the Sequence Read Archive (SRA) under SRA accession numbers SRR23032921 and SRR23032922.
